# Utility of PHQ-2, PHQ-8 and PHQ-9 for detecting major depression in primary health care: a validation study in Spain

**DOI:** 10.1017/S0033291722002835

**Published:** 2023-09

**Authors:** Irene Gómez-Gómez, Isabel Benítez, Juan Bellón, Patricia Moreno-Peral, Bárbara Oliván-Blázquez, Ana Clavería, Edurne Zabaleta-del-Olmo, Joan Llobera, Maria J. Serrano-Ripoll, Olaya Tamayo-Morales, Emma Motrico

**Affiliations:** 1 Department of Psychology, Universidad Loyola Andalucía, Dos Hermanas, Seville, Spain; 2 Prevention and Health Promotion Research Network (redIAPP)/Network for Research on Chronicity, Primary Care, and Health Promotion (RICAPPS), Barcelona, Spain; 3 Department of Methodology of Behavioral Sciences, Universidad de Granada, Granada, Spain; 4 Biomedical Research Institute of Málaga (IBIMA), Málaga, Spain; 5 El Palo Health Centre, Andalusian Health Service (SAS), Málaga, Spain; 6 Department of Public Health and Psychiatry, University of Málaga (UMA), Málaga, Spain; 7 Department of Psychology and Sociology, Universidad de Zaragoza, Zaragoza, Spain; 8 Institute for Health Research Aragón (IISA), Zaragoza, Spain; 9 Primary Care Research Unit, Área de Vigo, SERGAS, Vigo, Spain; 10 I-Saúde Group, Galicia Sur Health Research Institute (IIS Galicia Sur), SERGAS-UVIGO, Vigo, Spain; 11 Fundació Institut Universitari per a la recerca a l'Atenció Primària de Salut Jordi Gol i Gurina (IDIAPJGol), Barcelona, Spain; 12 Atenció Primària Barcelona Ciutat, Gerència Territorial de Barcelona, Institut Català de la Salut, Barcelona, Spain; 13 Nursing department, Faculty of Nursing, Universitat de Girona, Girona, Spain; 14 Primary Care Research Unit of Mallorca, Balearic Islands Health Services, Palma de Mallorca, Spain; 15 Health Research Institute of the Balearic Islands (IdISBa), Palma de Mallorca, Spain; 16 Unidad de Investigación en Atención Primaria de Salamanca (APISAL), Instituto de Investigación Biomédica de Salamanca (IBSAL), Salamanca, Spain

**Keywords:** Diagnostic accuracy, major depression, patient health questionnaire, primary health care, validity study

## Abstract

**Background:**

Primary health care (PHC) professionals may play a crucial role in improving early diagnosis of depressive disorders. However, only 50% of cases are detected in PHC. The most widely used screening instrument for major depression is the Patient Health Questionnaire (PHQ), including the two-, eight- and nine-item versions. Surprisingly, there is neither enough evidence about the validity of PHQ in PHC patients in Spain nor indications about how to interpret the total scores. This study aimed to gather validity evidence to support the use of the three PHQ versions to screen for major depression in PHC in Spain. Additionally, the present study provided information for helping professionals to choose the best PHQ version according to the context.

**Methods:**

The sample was composed of 2579 participants from 22 Spanish PHC centers participating in the EIRA-3 study. The reliability and validity of the three PHQ versions for Spanish PHC patients were assessed based on responses to the questionnaire.

**Results:**

The PHQ-8 and PHQ-9 showed high internal consistency. The results obtained confirm the theoretically expected relationship between PHQ results and anxiety, social support and health-related QoL. A single-factor solution was confirmed. Regarding to the level of agreement with the CIDI interview (used as the criterion), our results indicate that the PHQ has a good discrimination power. The optimal cut-off values were: ⩾2 for PHQ-2, ⩾7 for PHQ-8 and ⩾8 for PHQ-9.

**Conclusions:**

PHQ is a good and valuable tool for detecting major depression in PHC patients in Spain.

## Introduction

Depression is a common mental disorder affecting a high percentage of the population. In 2019, 5.02% of the world population suffered from depression (Institute of Health Metrics & Evaluation, 2019), with this rate having increased considerably during the COVID-19 pandemic (Bueno-Notivol et al., 2021). PHC professionals may play a crucial role in improving early diagnosis and management of depressive disorders for two reasons. Firstly, major depression is the mental disorder with the highest prevalence (around 10%) among Primary Health Care patients (PHC) (Craven & Bland, 2013; Serrano-Blanco et al., 2010). Secondly, around 83% of the population had used PHC services over the last 12 months (Macinko, de Andrade, de Souza Junior, & Lima-Costa, 2019). Spain is the country with the largest volume of PHC visits in Europe. In Spain, PHC is organized into around 3000 PHC centers (Government of Spain, 2021). However, it has been estimated that only 22–31% of patients with major depression received a correct diagnosis in Primary Health Care (Aznar-Lou et al., 2018; Fernández et al., 2010). Having in mind this scenario and the current situation of saturation of mental health services, it is crucial that PHC professionals are provided with the instruments necessary to detect depression in PHC patients (Ferenchick, Ramanuj, & Pincus, 2019).

Among the available tools, semi-structured or structured interviews are the most accurate instruments for detecting depression (Brugha, Bebbington, & Jenkins, 1999). However, this is a time-consuming tool not useful in routine PHC practice. In addition, the final diagnosis may vary according to the screening instrument employed. Thus, according to the results reported by Levis et al. (2018), patients undergoing the Mini-International Neuropsychiatric Interview (MINI) (Sheehan et al., 1998) were more likely to receive a diagnosis of major depression than those who underwent the Composite International Diagnostic Interview (CIDI) (WHO, 1997), although the CIDI provided a deeper diagnosis of depression.

With regard to questionnaires, the most widely used screening tool for detecting major depression in PHC is the Patient Health Questionnaire (PHQ) (Ferenchick et al., 2019; Maurer, Raymond, & Davis, 2018). The PHQ is derived from the *New Procedure for Diagnosing Mental Disorders in Primary Care* (PRIME-MD study), which was originally developed to detect depression, anxiety, alcohol abuse, somatoform disorder, and eating disorders in PHC (Spitzer et al., 1994). The PHQ was originally a nine-item questionnaire that was developed to assess major depression in PHC patients in the UK, showing adequate internal consistency (*α* = 0.89; Kroenke, Spitzer, and Williams, 2001). According to the authors, total scores can be interpreted using two different strategies: a cut-off value, which determines whether the person has or not a diagnosis of depression, or a diagnostic algorithm, which requires a score ⩾ 2 in five items, including item 1 or 2. However, according to previous studies, the algorithm has poorer psychometric properties (pooled sensitivity 35%, pooled specificity 95%), as compared to the PHQ-9 cut-off point ⩾ 10 (He et al., 2020).

Since PHQ-9 was created, many studies, including systematic reviews and meta-analyses, have been published to evaluate its utility from different perspectives (Aslan et al., 2020; Costantini et al., 2021; Diez-Quevedo, Rangil, Sanchez-Planell, Kroenke, & Spitzer, 2001; Gelaye et al., 2013; He et al., 2020; Levis et al., 2017, 2020; Manea, Gilbody, & McMillan, 2012; Muñoz-Navarro et al., 2017; Wu et al., 2020). Most studies were aimed at establishing the optimal cut-off point for detecting major depression. For instance, an individual participant data meta-analysis showed that the most widely used cut-off point (PHQ-9 ⩾10) had a pooled sensitivity of 67% and a pooled specificity of 86% (He et al., 2020). However, evidence about the validity of these cut-off values is not consistent. A meta-analysis based on 18 validation studies did not reveal substantial differences in the pooled sensitivity and specificity of the different PHQ-9 cut-off points (8 to 11) when using different standardized interviews based on DSM or ICD-10 for diagnosis of depression as a criterion (Manea et al., 2012). In that sense, the authors of PHQ-9 recommend using a different cut-off point based on the population to be assessed (Kroenke, Spitzer, Williams, & Löwe, 2010). This recommendation is consistent with current consensus about validity, where validation is described as a continuous process of gathering evidence to support the interpretation of scores for the specific purposes of the test (American Educational Research Association, American Psychological Association, National Council on Measurement in Education, 2014).

Shorter versions of the PHQ-9 have also been used in PHC: PHQ-2 (Kroenke, Spitzer, & Williams, 2003) and PHQ-8 (Kroenke et al., 2009). PHQ-2 contains the first two items of PHQ-9 (depressed mood and anhedonia) and is a very brief prescreening tool. The PHQ-8 consists of the first eight items of the PHQ-9 (omitting the item about suicidal ideation) in an attempt to avoid the problems reported about the inaccuracy of this item in assessing suicide risk, especially in non-psychiatric populations (Razykov, Ziegelstein, Whooley, & Thombs, 2012; Walker et al., 2011). Both, PHQ-2 (cut-off >3: sensitivity 83%, specificity 92%) and PHQ-8 (cut-off ⩾10: sensitivity of 100%, specificity 95%) showed adequate validity evidence regarding the relationship with a criterion in the American population (Kroenke et al., 2003, 2009).

Despite the range of studies available on the utility of PHQ, few studies have explored the psychometric properties of the Spanish versions of PHQ-2, PHQ-8 and PHQ-9 in PHC in Spain. The Spanish version of PHQ-9 showed good sensitivity (84%) and specificity (92%) in hospitalized patients in Spain, as compared to the diagnosis established by a mental health professional (Diez-Quevedo et al., 2001). Two additional studies confirmed the utility of PHQ-9 in assessing depression in PHC when administered telephonically (Pinto-Meza, Serrano-Blanco, Peñarrubia, Blanco, & Haro, 2005), and when the Structured Clinical Interview for DSM Disorders (SCID-I) is used as a criterion (Muñoz-Navarro et al., 2017). The studies conducted in other Spanish-speaking populations in Latin America raise concern about linguistic and cultural differences (Aslan et al., 2020; Baader et al., 2012; Urtasun et al., 2019; Wulsin, Somoza, & Heck, 2002). Regarding PHQ-2, a study of Spanish pregnant women who attended in PHC concluded that PHQ-2 (cut-off point ⩾2) had a good sensitivity (84.5%) and specificity (79.5%), taking PHQ-9 as a criterion (Rodríguez-Muñoz et al., 2017). No studies were found assessing the psychometric properties of PHQ-8 in the Spanish population. Therefore, further validity evidence is needed to support the use of PHQ-2, PHQ-8 and PHQ-9 to assess major depression and establish the optimal cut-off points for PHC patients in Spain. This would allow PHC professionals identify patients at risk of depression using a rapid, easy-to-use method. The purpose of screening is to ensure the adequate management of depression, which is performed based on a stepped care and collaboration model between primary care and mental health services (Ministry of Health, Social Services & Equality, 2014).

This study aimed to gather validity evidence supporting the use of PHQ-2, PHQ-8 and PHQ-9 as a tool for assessing major depression in the Spanish PHC population. To such purpose, a validation study was conducted where different sources of validity were combined and integrated to evaluate whether or not the total scores obtained in the three PHQ versions could be interpreted to detect major depression in PHC.

## Methods

### Design and study setting

A cross-sectional multicenter study was carried out in 22 PHC centers in six regions of Spain. PHC centers were recruited in the context of the EIRA-3 study (Zabaleta-del-Olmo et al., 2021), a randomized controlled hybrid type II preventive trial conducted in 25 PHC centers from seven regions of Spain. The centers included in the EIRA-3 study were located in Andalusia (*n* = 2); the Basque country (*n* = 3); Aragon (*n* = 4); the Balearic Islands (*n* = 4); Castile and Leon (*n* = 4); Catalonia (*n* = 4) and; Galicia (*n* = 4). However, three PHC centers from the Basque country were excluded from the study since they did not screen for baseline depression using a CIDI interview.

### Participants

Participants were recruited from PHC centers between February 2017 and January 2018. Eligible participants included subjects aged 45–75 years with at least two lifestyle risk factors (low physical activity, smoking consumption and/or unhealthy diet). Participants were excluded if they had severe mental illness or cognitive impairment, advanced serious physical illness, were not autonomous for daily activities, were involved in a home health care program, were receiving cancer treatment or palliative care, and did not live in the area during the study.

A total of 4387 participants were assessed for eligibility. Of them, 69.9% (*n* = 3062) provided informed consent and met the inclusion criteria. In total, 2.5% of participants (*n* = 78) were excluded due to incomplete PHQ-9 responses and 13.2% (*n* = 405) due to incomplete CIDI responses. The final sample was composed of 2579 participants. The total sample was randomly divided into three subsamples to conduct separate analysis for PHQ-2, PHQ-8 and PHQ-9. Subsamples were composed of 859, 860 and 860 participants for PHQ-2, PHQ-8 and PHQ-9, respectively. For PHQ-8 and PHQ-9, the two latter subsamples were additionally and randomly divided into two groups for crossed validation; it is detailed below in the description of the analysis conducted for assessing dimensionality. Table 1 shows the sociodemographic characteristic of each subsample, which were not significantly across groups.

**Table 1.** Respondent characteristics

	PHQ-2 *n* = 859	PHQ-8 *n* = 860	PHQ-9 *n* = 860
Age (years), M (s.d.)	58.35 (7.9)	57.93 (8.2)	57.66 (8.1)
Sex, *n* (%)			
Male	371 (43.2)	387 (45.0)	412 (47.9)
Female	488 (56.8)	473 (55.0)	448 (52.1)
Marital Status, *n* (%)			
Married or living with partner	613 (71.4)	604 (7.4)	581 (67.7)
Separated or divorced	117 (13.6)	108 (12.6)	108 (12.6)
Widowed or single	128 (14.9)	146 (17.0)	168 (19.6)
Other	0 (0.0)	0 (0.0)	1 (0.1)
Missing	1 (0.1)	1 (0.1)	2 (0.2)
Work status, *n* (%)			
Employed	387 (45.2)	398 (46.4)	397 (46.3)
Retired	230 (26.8)	223 (26.0)	231 (26.9)
Unemployed	75 (8.8)	82 (9.6)	82 (9.6)
Looking after family or home	113 (13.2)	100 (11.7)	101 (11.8)
Other (leave of absence for work, incapacity for work etc.)	52 (6.1)	55 (6.4)	47 (5.5)
Education level, *n* (%)			
Lower than primary education	38 (4.4)	58 (6.8)	56 (6.5)
Primary education	363 (42.3)	317 (36.9)	301 (35.1)
Secondary education	318 (37.1)	346 (4.3)	334 (38.9)
College and above	139 (16.2)	138 (16.1)	167 (19.5)
Depression (HSCL-25), M (s.d.)	1.59 (0.52)	1.56 (0.46)	1.53 (0.46)
Anxiety (HSCL-25), M (s.d.)	1.52 (0.45)	1.51 (0.42)	1.53 (0.47)
Anxiety (GAD-7), M (s.d.)	4.26 (4.7)	4.03 (4.5)	3.92 (4.5)
Social support (DUKE- 11), M (s.d.)	45.38 (9.1)	45.85 (8.3)	45.55 (8.5)
HRQoL index (EQ-5D- 3L index), M (s.d.)	0.82 (0.2)	0.83 (0.2)	0.82 (0.2)

*Note**.*** *p < 0.05; **p < 0.01; ***p < 0.001.

### Procedure

Participants were recruited by PHC professionals during routine visits or by telephone, and by informative posters and local advertisements displayed in the waiting room of PHC centers. The participants who met the inclusion criteria and were interested in participating in the study read the information sheet and signed informed consent. Then, participants were invited telephonically to attend an assessment session. All data were collected all in once at baseline by local trained personnel, coordinated at central level, through a face-to-face interview (60 min approximately) which was recorded in an electronic data collection booklet specifically created for the EIRA-3 study.

The study protocol was approved by the Research Ethics Committee of the IDIAP Jordi Gol (approval number P16/025) and the local ethics committees of each participating Autonomous Communities. The protocol was registered at ClinicalTrials.gov, NCT03136211.

### Instruments

#### Sociodemographic data

The sociodemographic questionnaire collected information about sex, age, education level, marital status, and employment status.

#### PHQ-9

The PHQ-9 is a 9-item self-administered questionnaire (Kroenke et al., 2001; Kroenke & Spitzer, 2002) created according to the diagnostic criteria for major depression disorder proposed in the 4^th^ version of the Diagnostic and Statistical Manual of Mental Disorders (DSM-IV). PHQ-9 explores the presence of the symptoms described in Table 2 over the past two weeks. Each PHQ-9 item contains four Likert-response categories, ranging from 0 *‘Not at all’* to 3 *‘Nearly every day’.* The total score ranges between 0 to 27 points, with the original cut-off point set at ⩾10 to determine the presence of major depression (Kroenke et al., 2001). Higher scores indicate more depressive symptoms. McDonald's omega coefficient for the PHQ-9 was 0.89 in PHC Spanish patients (Muñoz-Navarro et al., 2017). PHQ-9 has also been proposed as a diagnostic tool based on a diagnostic algorithm (Kroenke et al., 2001).

**Table 2.** Descriptive statistics of the PHQ-2, PHQ-8 and the PHQ-9

	Minimum	Maximum	Mean	s.d.	Skewness	Kurtosis
PHQ-2						
Item 1	0	3	0.57	0.90	1.60	1.62
Item 2	0	3	0.64	0.91	1.41	1.06
Sum score	0	6	1.21	1.64	1.47	1.48
PHQ-8						
Item 1	0	3	0.52	0.86	1.69	1.98
Item 2	0	3	0.60	0.87	1.49	1.50
Item 3	0	3	0.75	1.06	1.19	0.00
Item 4	0	3	0.90	1.04	0.95	−0.32
Item 5	0	3	0.48	0.90	1.91	2.48
Item 6	0	3	0.34	0.72	2.42	5.43
Item 7	0	3	0.30	0.72	2.66	6.44
Item 8	0	3	0.23	0.64	3.23	10.21
Sum Score	0	24	4.11	4.63	1.71	3.12
PHQ-9						
Item 1	0	3	0.56	0.91	1.64	1.67
Item 2	0	3	0.62	0.91	1.47	1.26
Item 3	0	3	0.83	1.11	1.04	−0.39
Item 4	0	3	0.95	1.08	0.89	−0.53
Item 5	0	3	0.57	0.98	1.59	1.15
Item 6	0	3	0.35	0.75	2.33	4.79
Item 7	0	3	0.33	0.76	2.44	5.10
Item 8	0	3	0.29	0.70	2.73	6.97
Item 9	0	3	0.10	0.45	5.25	28.50
Sum Score	0	27	4.60	5.12	1.75	3.48

#### PHQ-2

The PHQ-2 consists of the first two items of PHQ-9 exploring the presence of depressed mood and anhedonia over the past two weeks, scoring from 0 ‘*not at all*’ to 3 ‘*nearly every day*’ (Kroenke et al., 2003). Total score ranges from 0 to 6. The Cronbach's alpha coefficient for the Spanish version was 0.71 in Colombian PHC patients (Scoppetta, Cassiani-Miranda, Arocha-Díaz, Cabanzo-Arenas, & Campo-Arias, 2021). The cut-off point ⩾ 3 has been proposed as the value confirming the presence of major depression (Manea et al., 2016).

#### PHQ-8

The PHQ-8 contains the first eight items of the PHQ-9 (Kroenke & Spitzer, 2002), exploring the presence of depressive symptoms over the past two weeks with each item scoring from 0 ‘*not at all*’ to 3 ‘*nearly every day*’. Total score ranges from 0 to 24 with a proposed cut-off point ⩾ 10 to detect major depression (Wu et al., 2020). Cronbach's alpha coefficient was 0.81 for outpatients recruited at three major public hospitals in Bolivia (Schantz et al., 2017) and 0.92 for the general population of Puerto Rican adults (Pagán-Torres, González-Rivera, & Rosario-Hernández, 2020).

#### The CIDI – Module E

The CIDI-module E is a standardized diagnostic interview created by the WHO to assesses major depression (WHO, 1997). The CIDI includes the first two screening questions assessing anhedonia and depressive mood during the last 12 months for a period of two consecutive weeks. When at least one of these two screening questions is affirmatively answered, 31 additional items are asked including both yes/no and free-text answers. According to a recent systematic review, the CIDI is the second most common diagnostic interview used in primary care to screen for depression using the PHQ-9 (Costantini et al., 2021). The CIDI showed excellent inter-rater reliability (> 0.90) in most of the diagnoses. Test-retest reliability reached good-to-excellent Kappa indexes for most of the CIDI modules, including the depression module (Wittchen, 1994). The CIDI-depression module showed an Area Under the Curve (AUC) of 0.75, a sensitivity of 53.3%, and a specificity of 93.7% using SCID as a criterion (Haro et al., 2006).

#### The 25-item version of the Hopkins Symptom Checklist (HSCL-25)

The HSCL-25 is a self-administered 25-item questionnaire (Nabbe et al., 2019) that assesses anxiety (items 1–10) and depression (items 11–25). Each item offers four Likert response options, ranging from 1 ‘*Not at all*’ to 4 ‘*Extremely*’. The total score is calculated by dividing the sum of the scores of all items by 25 (all items), 10 (anxiety dimension) or 15 (depression dimension); total scores range from 1 to 4 points. Cronbach's alpha coefficient was 0.92 in Spanish PHC patients (Rodríguez-Barragán et al., 2021).

#### Generalized Anxiety Disorder Scale (GAD-7)

The GAD-7 is a 7-item scale measuring symptoms of generalized anxiety (Spitzer, Kroenke, Williams, & Löwe, 2006). Each GAD-7 item contains four Likert-response options, ranging from 0 ‘*Not at all*’ to 3 ‘*Nearly every day*’. Total score ranges from 0 to 21 points. Higher scores indicate more symptoms of anxiety. Cronbach's alpha coefficient was 0.94 in the Spanish general population (Garcia-Campayo et al., 2010).

#### Functional social support questionnaire (DUKE-UNC-11)

The DUKE-UNC-11 is a multidimensional questionnaire assessing functional social support (Broadhead, Gehlbach, de Gruy, & Kaplan, 1988). The DUKE-UNC-11 consists of 11 items with five Likert-response options ranging from 1 *‘much less than I want’* to 5 *‘as much as I want’.* The total score ranges from 11 to 55 points, where higher scores indicate more functional social support. Cronbach's alpha coefficient was 0.92 for Spanish PHC patients (Bellón, Delgado Sánchez, Luna del Castillo, & Lardelli Claret, 1996).

#### Health-related Quality of Life (EQ-5D-3L)

The EQ-5D-3L is a multi-attribute instrument for assessing health-related quality of life (HR-QoL) (Rabin & De Charro, 2001; Szende, Oppe, & Devlin, 2007). EQ-5D-3L evaluates problems in different dimensions (mobility, self-care, daily activities, pain/discomfort, and anxiety/depression) and self-rated health status. We used a single summary HR-QoL index calculated based on a scoring algorithm for the Spanish population (EuroQol Research Foundation, 2018; Szende et al., 2007).

### Statistical analysis

All statistical analyses were carried out using the statistical package SPSS (V 26) and JASP software (V 0.14.1.0). To evaluate the psychometric properties of PHQ-2, PHQ-8 and PHQ-9, the total sample was randomly divided into three subsamples. The internal consistency of PHQ-8 and PHQ-9 scores was assessed according to McDonald's omega coefficient and the 95% confidence intervals (CI). Validity evidence based on internal structure and on relations to other variables was collected following the Standards for Educational and Psychological Testing guidelines (AERA et al., 2014). First, the dimensionality of the instrument was explored by conducting an exploratory factor analysis (EFA) and a confirmatory factor analysis (CFA). For such purpose, the PHQ-8 and PHQ-9 subsamples were randomly divided into two with the same number of participants to perform crossed validation among samples. Kaiser-Meyer-Olkin (KMO) and Bartlett's tests were used to assess the appropriateness of applying EFA to PHQ-8 and PHQ-9 data sets. Principal Axis Factoring extraction method and Varimax rotation were used to perform EFA. Parallel analysis was used to decide the number of factors to be extracted. Regarding CFA, it was performed using the maximum likelihood method. The Goodness of Fix Index (GFI), the Comparative Fit Index (CFI), and the Root Mean Square Error of Approximation (RMSEA) were used to estimate the goodness-of-fit of the model. Values of GFI and CFI higher than 0.90, and of RMSEA lower than 0.05 were considered appropriate (Hu & Bentler, 1999; McDonald & Ho, 2002). Measurement invariance was assessed by multi-group analysis, where participants were compared according to variables previously described in the literature as potential sources of bias when using PHQ. Specifically, the structure of the construct was compared across groups according to sex, age and educational level (Bellón et al., 2011; González-Blanch et al., 2018; Patel et al., 2019). To assess invariance between groups, the change in chi-square value (Δχ^2^) and its *p* value, CFI (ΔCFI) and RMSEA (ΔRMSEA) values were calculated. Four models were tested sequentially, from the least to the most restrictive level of invariance (configural, metrics, strong and, strict). Invariance between groups was settled when the *p* value of Δχ^2^ was non-significant, and when the change of the RMSEA and CFI values was lower than 0.015 and 0.01 respectively (Chen, 2007). Associations with other variables were explored by: (i) obtaining evidence about convergence between variables by calculating correlations between the three versions of the PHQ and the theoretically related variables (HCLS-25, GAD-7, DUKE-UNC-11 and EQ-5D-3); and (ii) analyzing the level of agreement between the scores obtained in the three versions of the PHQ and in the criterion (the CIDI) through Receiver Operating Characteristic (ROC) curve analysis. We expected a positive correlation between the different PHQ versions and the instrument measuring similar constructs (HCLS-25 and GAD-7), as well as a negative correlation between PHQ versions and the other instruments measuring related but distinct variables (DUKE-UNC-11 and EQ-5D-3). AUC was calculated and interpreted as follows: 0.5–0.6 ‘*no discrimination*’; 0.6−0.7 ‘*low discrimination*’; 0.7–0.8 ‘*acceptable discrimination*’; 0.8–0.9 ‘*good discrimination*’; > 0.9 ‘*excellent discrimination*’ (Muñiz, 2018). Moreover, sensitivity rates, specificity rates, positive and negative likelihood ratios, and positive and negative predictive values were extracted. Youden's index was calculated to determine the optimal cut-off value for a good sensitivity/specificity balance (Youden, 1950). Convergence between the PHQ-9 diagnostic algorithm and the optimal PHQ-9 cut-off according to Youden's index was calculated using Cohen's kappa coefficient index (Cohen, 1960).

## Results

### Descriptive statistics and internal consistency

Table 2 shows descriptive statistics for PHQ-2, PHQ-8 and PHQ-9. McDonald's omega coefficient (*ω*) was 0.83 (95% CI 0.81–0.84) for the PHQ-8 and 0.84 (95% CI 0.82–0.85) for the PHQ-9. The items in the two versions showed discrimination indexes above the established minimum, which was 0.30.

### Validity evidence based on internal structure for PHQ-8 and PHQ-9

KMO (greater than 0.7) and Bartlett's test (*p* < 0.001) indicated the adequacy of applying EFA to PHQ-8 and PHQ-9. Regarding AFE, a one-factor solution was extracted based on parallel analysis calculated over the first half of each subsample (see Table 3). Using the second half of each subsample, the one-factor model showed a reasonable fit of the model to the data for both PHQ-8 (GFI = 0.936; CFI = 0.899; RMSEA = 0.10) and PHQ-9 (GFI = 0.947; CFI = 0.932; RMSEA = 0.08). Regarding to multi-group analysis by sex, age and educational level, results confirmed invariance between groups in all cases, at least at configural level (see online Supplementary Tables A1 and A2 in supplementary material). It is worth noting that χ^2^ becomes usually significant due to its sensitivity to sample size.

**Table 3.** Factor loadings derived from EFA for the PHQ-8 and the PHQ-9

	PHQ-8	PHQ-9
Items	Factor's loading^a^	Factor's loading^a^
Depressed mood (item 1)	0.81	0.73
Anhedonia (item 2)	0.79	0.77
Sleep problems (item 3)	0.46	0.51
Feelings of tiredness (item 4)	0.68	0.64
Changes in appetite (item 5)	0.58	0.47
Feelings of guilt or worthlessness (item 6)	0.74	0.69
Difficult to focus (item 7)	0.64	0.65
Feelings of slowness or concern (item 8)	0.57	0.51
Suicidal ideation (item 9)	–	0.55

a Based on parallel analysis.

### Convergence between PHQ-2, PHQ-8 and PHQ-9 and related variables

The postulated relationship between the three PHQ versions and theoretically related variables was confirmed. Correlation between the three versions and both HSCL-25 and GAD-7 were positive (⩾ 0.50) and significant (see Table 4). Correlations with DUKE-UNC-11 and HR-QoL index were negative (ranging from −0.30 to −0.50) and significant. PHQ-2 was the version with the highest correlation with theoretically related variables.

**Table 4.** Correlations between the PHQ-2, the PHQ-8 & the PHQ-9 and other variables measured thought the HSCL-25, the GAD-7, the DUKE-UNC-11 & the HRQoL index

	1	2	3
	*r*	*r*	*r*
1. Depression (PHQ-2)	–		
2. Depression (PHQ-8)	–	–	
3. Depression (PHQ-9)	–	–	–
4. Depression (HSCL-25 – depression dimension)	0.802***	0.799***	0.731***
5. Anxiety (HSCL-25 – anxiety dimension)	0.678***	0.651***	0.611***
6. Anxiety (GAD-7)	0.645***	0.613***	0.664***
7. Social support (DUKE-UNC-11)	−0.466***	−0.361***	−0.399***
8. Health-related QoL (HRQoL index)	−0.531***	−0.464***	−0.508***

*Note***.** *p < 0.05; **p < 0.01; ***p < 0.001. Since the total sample was divided into subsamples, correlations between scores in the other variables measured are not included.

### Validity evidence on the relationships with the gold-standard CIDI

According to the CIDI, the prevalence of major depression in the last 12 months was 4.8% (*n* = 123) for the total sample, 5.1% (*n* = 44) for the PHQ-2 subsample, 5.1% (*n* = 44) for the PHQ-8 subsample and 4.1% (*n* = 35) for the PHQ-9 subsample.

ROC curve analysis showed that PHQ-2, PHQ-8 and PHQ-9 had an AUC of 0.85 (95% CI 0.816–0.889; s.e. = 0.019; *p* < 0.001); 0.90 (95% CI 0.846–0.953; s.e. = 0.027; *p* < 0.001) and 0.91 (95% CI 0.874–0.949; s.e. = 0.019; *p* < 0.001) respectively, which indicates good discrimination (see online Supplementary material).

Table 5 shows sensitivity, specificity, Youden's index, positive and negative likelihood ratios, and positive and predictive value of different cut-off scores for PHQ-2, PHQ-9 and PHQ-8, as compared against the CIDI. According to Youden's index, the optimal cut-off values were ⩾ 2 (*J* = 0.57) for PHQ-2, with a sensitivity of 88% and a specificity of 70%; ⩾ 7 (*J* = 0.68) for PHQ-8, with a sensitivity of 86% and a specificity of 81%; and ⩾ 8 (*J* = 0.68) for PHQ-9, with a sensitivity of 86% and a specificity of 82%.

**Table 5.** Sensitivity, specificity, Youden's index, likelihood ratios and predictive values at different cut-off scores of the PHQ-2, PHQ-9 and PHQ-8 when compared to the CIDI

Threshold Score for PHQ-2	Sensitivity	Specificity	Youden's index	LR +	LR−	PPV	NPV
⩾ 1	0.94	0.53	0.47	2.00	0.12	0.10	1.00
⩾ 2	**0.88**	**0.70**	**0.57**	**2.89**	**0.18**	**0.13**	**0.99**
⩾ 3	0.68	0.85	0.54	4.68	0.37	0.19	0.98
⩾ 4	0.55	0.92	0.47	6.63	0.49	0.24	0.97
**Threshold Score for PHQ-8**	**Sensitivity**	**Specificity**	**Youden's index**	**LR +**	**LR-**	**PPV**	**NPV**
⩾ 5	0.89	0.70	0.59	2.96	0.16	0.14	0.99
⩾ 6	0.89	0.76	0.65	3.74	0.15	0.17	0.99
⩾ 7	**0.86**	**0.81**	**0.68**	**4.65**	**0.17**	**0.20**	**0.99**
⩾ 8	0.80	0.85	0.65	5.47	0.24	0.23	0.99
⩾ 9	0.73	0.89	0.62	6.84	0.31	0.27	0.98
⩾ 10	0.70	0.91	0.62	7.89	0.32	0.30	0.98
⩾ 11	0.66	0.93	0.59	9.80	0.37	0.35	0.98
⩾ 12	0.64	0.95	0.59	13.70	0.38	0.42	0.98
⩾ 13	0.59	0.96	0.55	15.59	0.43	0.46	0.98
⩾ 14	0.52	0.97	0.50	19.44	0.49	0.51	0.97
⩾ 15	0.48	0.98	0.46	21.69	0.53	0.54	0.97
**Threshold Score for PHQ-9**	**Sensitivity**	**Specificity**	**Youden's index**	**LR +**	**LR−**	**PPV**	**NPV**
⩾ 5	0.94	0.64	0.59	2.65	0.09	0.10	1.00
⩾ 6	0.91	0.72	0.63	3.21	0.12	0.12	0.99
⩾ 7	0.86	0.78	0.64	3.89	0.18	0.14	0.99
⩾ 8	**0.86**	**0.82**	**0.68**	**4.81**	**0.17**	**0.17**	**0.99**
⩾ 9	0.80	0.86	0.66	5.55	0.23	0.19	0.99
⩾ 10	0.74	0.89	0.63	6.73	0.29	0.22	0.99
⩾ 11	0.69	0.90	0.59	7.16	0.35	0.23	0.99
⩾ 12	0.66	0.92	0.58	8.47	0.37	0.26	0.98
⩾ 13	0.60	0.94	0.54	9.90	0.43	0.30	0.98
⩾ 14	0.51	0.95	0.46	1.10	0.51	0.30	0.98
⩾ 15	0.49	0.96	0.44	11.45	0.54	0.33	0.98

*Note.* LR+, Positive Likelihood Ratio; LR−, Negative Likelihood Ratio; PPV, Positive Predictive Value; PPN, Negative Predictive Value. Bold shade indicate optimal cut-off value according to Youden´s index for PHQ-2, PHQ-8 and PHQ-9.

### Inconsistencies between PHQ-9 cut-off points and the CIDI

There were five participants with major depression according to the CIDI and PHQ-9 scores lower than 6. Exploration of these inconsistencies indicated that differences in final diagnosis derived from two situations: participants who answered ‘*not at all*’ to a specific item in PHQ-9 but ‘*yes*’ to the equivalent item in CIDI, i.e. participants who were not consistent in their responses; and participants who answered ‘*several days*’ to a specific item of PHQ-9 and ‘*yes*’ in the equivalent items of the CIDI. To confirm the conclusions formulated, analyses were replicated by excluding participants with inconsistent diagnoses. Results did not change for the PHQ-9 except for ROC analysis, where the AUC increased to 0.95 (95% CI 0.931–0.970; s.e. = 0.010; *p* < 0.0001). According to Youden's index, the optimal cut-off value was the same (PHQ-9 ⩾8) but sensitivity increased (*J* = 0.82; sensitivity 100%; specificity 82%).

### The PHQ-9 diagnostic algorithm

The scores obtained from the PHQ-9 diagnostic algorithm indicated a fair convergence with the CIDI (Cohen *κ* = 0.36; 95% CI 0.193–0.527) and with the proposed cut-off value for PHQ-9 (⩾ 8) (Cohen *κ* = 0.40; 95% CI 0.300–0.495). When the CIDI was used as a criterion, the algorithm showed an AUC of 0.72 indicating acceptable discrimination (95% CI 0.615–0.828; s.e. = 0.054, *p* < 0.001), with a sensitivity of 49% and a specificity of 96%. When the algorithm was compared with the cut-off value ⩾ 8, the AUC was 0.65 (95% CI 0.596–0.698; s.e. = 0.026, *p* < 0.001) indicating low discrimination, low sensitivity (29%), and adequate specificity (71%).

## Discussion

To the best of our knowledge, this is the first study to gather validity evidence supporting the use of PHQ-2, PHQ-8, and PHQ-9 as a tool to detect major depression in the Spanish PHC population. The present study suggests the optimal cut-off value for a good sensitivity/specificity balance were ⩾ 2 for PHQ-2, ⩾7 for PHQ-8 and ⩾8 for PHQ-9.

Considering the information provided by the CIDI, the prevalence of major depression in our sample was 4.8%, which is consistent with a recent study in PHC attendees in Spain (Vieta et al., 2021) but lower than rates found in other studies focused on PHC (Craven & Bland, 2013; Serrano-Blanco et al., 2010). However, as the aim of the present study was to collect validity evidence to support the use of PHQ, we cannot formulate conclusions about the prevalence, which will be addressed in future research studies.

In terms of psychometric properties, the PHQ-8 and the PHQ-9 showed high reliability as measured by the internal consistency of their scores, which is consistent with the results of previous studies assessing the validity of PHQ-9 in PHC in Spain (Muñoz-Navarro et al., 2017) and other Spanish-speaking countries from Latin America (Aslan et al., 2020; Schantz et al., 2017; Scoppetta et al., 2021).

Regarding validity evidence based on internal structure, a single-factor solution structure was supported, which is in agreement with previous studies in PHC patients in Spain (González-Blanch et al., 2018) and other Spanish-speaking populations (Alpizar, Laganá, Plunkett, & French, 2018; Aslan et al., 2020; Pagán-Torres et al., 2020). The one-factor solution was suggested in the exploratory phase of the analysis and confirmed in the confirmatory phase, where it showed reasonable goodness-of-fit indexes of the model in relation to data for both the PHQ-8 and PHQ-9. These results are similar to those reported in previous studies (González-Blanch et al., 2018; Patel et al., 2019). In addition, invariance across groups divided by sex, age and educational was confirmed, as it occurred in previous studies (González-Blanch et al., 2018; Patel et al., 2019). Validity evidence based on relationships with other variables confirmed the expected results, i.e. positive and significant relationships with anxiety and depression, and negative and significant associations with social support and H-RQoL. Additionally, ROC curve analysis allowed to establish, with good discrimination values, the optimal cut-off point using the CIDI as the criterion. According to the results, the optimal cut-off value for PHQ-2 is ⩾ 2, detecting 88% of PHC patients with major depressive disorders. Although the original validation study recommended a cut-off point ⩾ 3 (sensitivity 86%, specificity 90%), the authors used in that case an independent structured mental health professional interview as a criterion (Kroenke et al., 2003). In contrast, previous studies establish ⩾ 2 as the optimal cut-off values as well (Arroll et al., 2010; Rodríguez-Muñoz et al., 2017). In the present study, the originally proposed cut-off point (⩾3) had lower sensitivity (68%) and specificity (85%), as compared to the cut-off point of the original validation study. Thus, the cut-off point ⩾ 2 might be more effective in detecting major depression in the PHC setting, considering the CIDI as the criterion.

With regard to PHQ-8, the optimal cut-off point in the present study was ⩾ 7 (sensitivity 86%, specificity 81%). This cut-off point detects 86% of cases of major depressive disorders. A recent meta-analysis based on fully-structured interviews as the criterion proposed the cut-off points of ⩾ 7 and ⩾ 8 as the values with the optimal sensitivity/specificity balance (Wu et al., 2020). Using the CIDI as the criterion, Arroll et al. (2010) also pointed to the need of considering a cut-off value lower than 10.

Regarding the PHQ-9 diagnostic algorithm, our results are in line with previous studies revealing high specificity but low sensitivity (Manea, Gilbody, & McMillan, 2015). In that case, results also converge with previous findings of the present study and suggest lower cut-off values than in studies conducted by other authors. This could be explained by the criterion used: the CIDI. According to previous studies, the CIDI yields a deeper diagnosis of major depression compared to the MINI interview, which diagnoses twice as many cases of major depression than the CIDI (Levis et al., 2018). Taking into account that PHQ is used to detect major depression in clinical practice, the cut-off values employed must maximize sensitivity with an adequate specifity.

The study has several strengths. First, the large size and heterogeneity of the sample, as participants were recruited from diverse PHC centers over the country, which guarantees the representativeness of the sample. Second, a rigorous methodology was used based on the Standards for Educational and Psychological Testing (AERA et al., 2014). Third, it should be emphasized that participants were evaluated by an external unit and that all instruments were administered the same day. Regarding limitations, on the one hand, participants were recruited in the context of the EIRA-3 study, which is focused on contacting participants with unhealthy behaviors. Thus, our study sample differs from that used in the validation study of the original version of the PHQ-9 (Kroenke et al., 2001) in two main aspects. The first one is co-occurrence of unhealthy behaviors, and the second the age of participants, which were older in our study (45–75). Although this could have increased the probability that patients suffered from depression, the prevalence of depression was similar or even lower than expected (Institute of Health Metrics & Evaluation, 2019). In addition, the health behaviors evaluated are the most common in PHC, so participants were representative of the Spanish PHC population (Government of Spain, 2021; Gómez-Gómez et al., 2020). However, the scope of our study goes beyond estimating the prevalence of depression. Thus, this study also aimed at assessing the utility of the PHQ and providing evidence supporting its utility by showing its convergence with CIDI results. Hence, diagnoses established based on the CIDI were generally confirmed by the PHQ, independently of the characteristics and/or age of patients (which potential influence, if exists, would be true and not a bias as shown by invariance results). On the other hand, the instruments were administered by adjusting the model to the specific needs of each PHC center, using both, self-administered questionnaires and face-to-face interviews.

### Implications for practice

Having in mind the results of the study, and considering both, the adequate psychometric properties of the three versions and the context of the Spanish health system, where the opportunities of assessment are limited, our recommendations are: (1) using PHQ-2 with a cut-off point ⩾ 2 as a pre-screening instrument; (2) when PHQ-2 indicates major depression, applying PHQ-8 (with a cut-off point ⩾ 7) or PHQ-9 (with a cut-off point ⩾ 8). The decision of using PHQ-8 or PHQ-9 should be made considering that the only difference between them is item 9, which was created to assess suicidal ideation and self-harm. In the psychiatric setting, item 9 is used as an indicator of suicide risk (Wu et al., 2020), as it is considered a strong predictor of suicide attempt in psychiatry practice (Simon et al., 2016). However, several studies have found that it overestimates suicide risk in different populations and settings (Na et al., 2018; Razykov et al., 2012, 2013; Suarez et al., 2015; Walker et al., 2011). For instance, a study conducted in 841 patients with depression revealed that item 9 detected almost three times more patients at risk of suicide (41.1%) than a specific scale created to assess suicide risk (13.4%) (Na et al., 2018). Similar results were found in non-psychiatric patients such as veterans attended in PHC (Corson, Gerrity, & Dobscha, 2004) and in patients with medical conditions such as heart disease (Razykov et al., 2012; Suarez et al., 2015); cancer (Walker et al., 2011); and systemic sclerosis (Razykov, Hudson, Baron, & Thombs, 2013). In all these studies, only a small proportion of participants responding affirmatively to item 9 had suicidal ideation or planned to commit suicide. Considering that item 9 has a high rate of false positives, especially in non-psychiatric populations, it can be concluded that PHQ-8 is more effective in assessing major depression in non-psychiatric settings, in large population surveys or large epidemiological studies in which the study staff or the researchers cannot cope with affirmative responses, and in clinical and research settings where follow-up of positive responses may be delayed (Kroenke et al., 2010). However, PHQ-9 would be an adequate choice on suspicion of suicidal ideation in a personal evaluation where the professional could intervene somehow. Finally, we do not recommend the PHQ-9 diagnostic algorithm to be used to assess major depression in the Spanish PHC setting as it only detects 29 to 49% of cases of major depression.

Based on the results of the present study, it can be concluded that PHQ is a good and valuable tool for screening and assessing major depression in PHC patients in Spain. Having adequate tools for detecting major depression could contribute to the early detection, implementation of prevention policies and programs, and administration of adequate and early treatments. Future studies could use a mixed methods approach to collect qualitative evidence related to the response processes developed by participants to respond the questionnaire.

## Supplementary material

For supplementary material accompanying this paper visit https://doi.org/10.1017/S0033291722002835. click here to view supplementary material
