# Supplementary material for: Utility of PHQ-2, PHQ-8 and PHQ-9 for detecting major depression in primary health care: a validation study in Spain
Source: Psychol Med. 2022 Oct 19;53(12):5625–35. doi: 10.1017/S0033291722002835 (PMC10482708; doi:10.1017/S0033291722002835)
Supplement: Supplementary file 1 [file S0033291722002835sup001.docx]

**Other supplementary material**

**
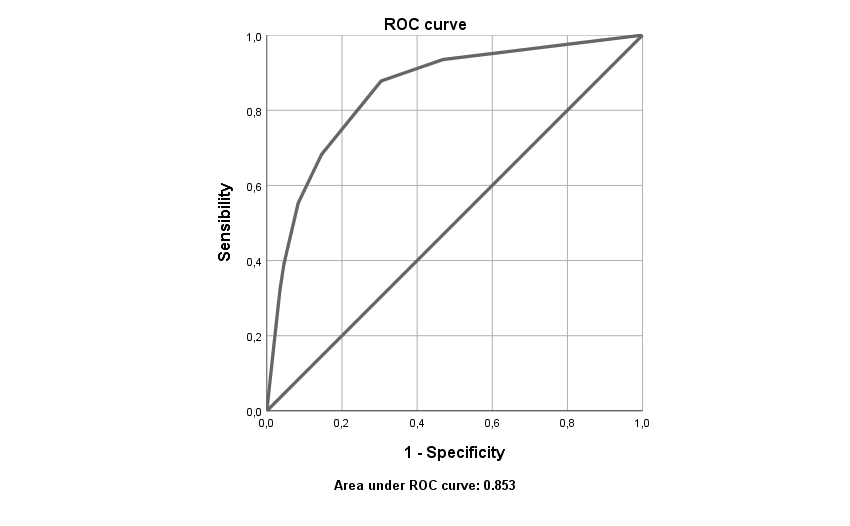
**

**Figure A1. ROC curve for the PHQ-2**


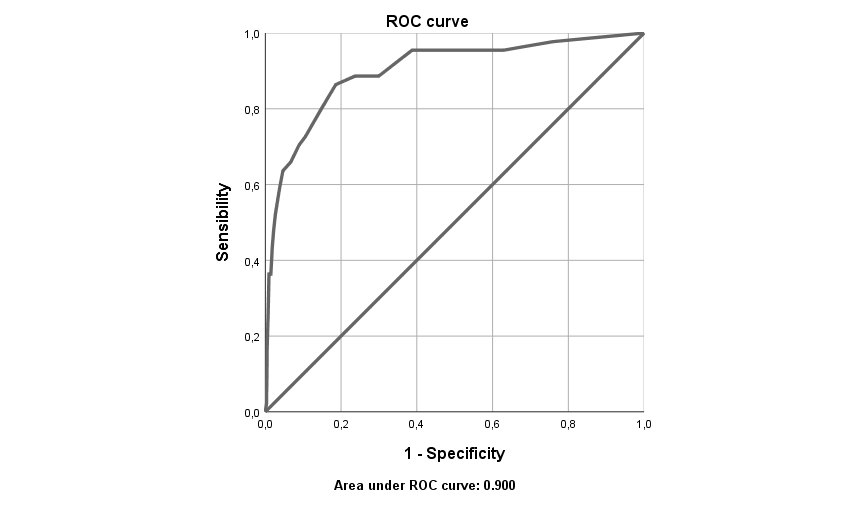


**Figure A2. ROC curve for the PHQ-8**


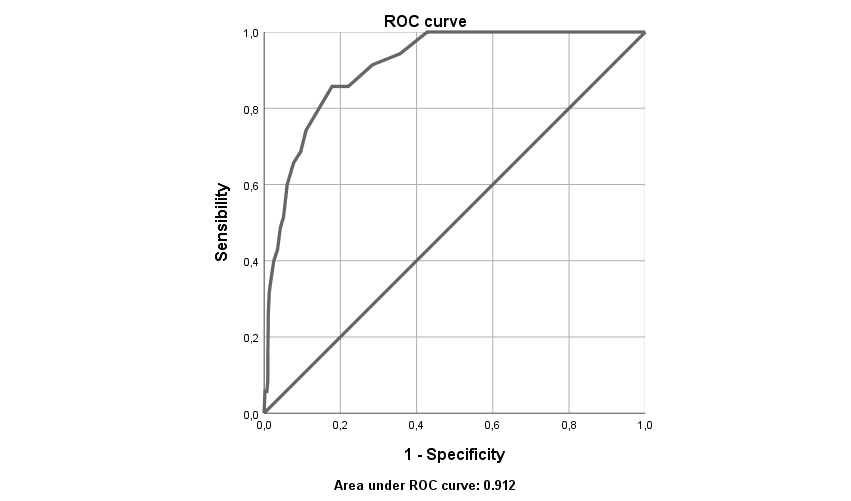


**Figure A3. ROC curve for the PHQ-9**

**Table A1**

*Goodness-of-fit indices for PHQ-8 from multi-group invariance analyses across sex, age and educational level.*

|  |  | **χ2** | **(df)** | **Δχ2** | **p value** | **RMSEA** | **ΔRMSEA** | **CFI** | **TLI** | **ΔCFI** |
| --- | --- | --- | --- | --- | --- | --- | --- | --- | --- | --- |
| **Sex** | Configural | 186.846 | 40 |  |  | .074 |  | .890 | .846 |  |
|  | Metric | 188.487 | 47 | 1.641 | .977 | .067 | -.007 | .894 | .874 | .004 |
|  | Strong | 192.435 | 55 | 3.948 | .862 | .061 | -.006 | .897 | .895 | .003 |
|  | Strict | 196.866 | 64 | 4.431 | .881 | .056 | -.005 | .901 | .913 | .004 |
|  |  | **χ2** | **(df)** | **Δχ2** | **p value** | **RMSEA** | **ΔRMSEA** | **CFI** | **TLI** | **ΔCFI** |
| **Age** | Configural | 163.323 | 60 |  |  | .064 |  | .882 | .835 |  |
|  | Metric | 190.722 | 74 | 27.399 | .017 | .061 | -.003 | .867 | .849 | -.015 |
|  | Strong | 206.081 | 90 | 15.359 | .499 | .055 | -.006 | .867 | .876 | .000 |
|  | Strict | 235.225 | 108 | 29.144 | .047 | .053 | -.002 | .855 | .887 | -.012 |
|  |  | **χ2** | **(df)** | **Δχ2** | **p value** | **RMSEA** | **ΔRMSEA** | **CFI** | **TLI** | **ΔCFI** |
| **Educational level** | Configural | 239.516 | 60 |  |  | .066 |  | .872 | .821 |  |
|  | Metric | 255.195 | 74 | 15.679 | .333 | .06 | -.006 | .871 | .854 | -.001 |
|  | Strong | 261.578 | 90 | 6.383 | .983 | .053 | -.007 | .878 | .886 | .007 |
|  | Strict | 305.787 | 108 | 44.209 | .001 | .052 | -.001 | .859 | .891 | -.019 |

**Table A2**

*Goodness-of-fit indices for PHQ-9 from multi-group invariance analyses across sex, age and educational level.*

|  |  | **χ2** | **(df)** | **Δχ2** | **p value** | **RMSEA** | **ΔRMSEA** | **CFI** | **TLI** | **ΔCFI** |
| --- | --- | --- | --- | --- | --- | --- | --- | --- | --- | --- |
| **Sex** | Configural | 123.349 | 54 |  |  | .055 |  | .935 | .913 |  |
|  | Metric | 169.578 | 62 | 46.229 | ≥.0001 | .064 | .009 | .899 | .882 | -.036 |
|  | Strong | 188.488 | 71 | 18.91 | .0260 | .062 | -.002 | .889 | .888 | -.01 |
|  | Strict | 258.755 | 81 | 70.267 | ≥.0001 | .072 | .010 | .833 | .851 | -.056 |
|  |  | **χ2** | **(df)** | **Δχ2** | **p value** | **RMSEA** | **ΔRMSEA** | **CFI** | **TLI** | **ΔCFI** |
| **Age** | Configural | 285.509 | 81 |  |  | .051 |  | .921 | .895 |  |
|  | Metric | 297.382 | 97 | 11.873 | .753 | .046 | -.005 | .923 | .914 | .002 |
|  | Strong | 304.85 | 115 | 7.468 | .986 | .042 | -.004 | .927 | .931 | .004 |
|  | Strict | 331.04 | 135 | 26.19 | .160 | .039 | -.003 | .924 | .940 | -.003 |
|  |  | **χ2** | **(df)** | **Δχ2** | **p value** | **RMSEA** | **ΔRMSEA** | **CFI** | **TLI** | **ΔCFI** |
| **Educational level** | Configural | 216.005 | 81 |  |  | .063 |  | .889 | .852 |  |
|  | Metric | 242.187 | 97 | 26.182 | .052 | .059 | -.004 | .881 | .867 | -.008 |
|  | Strong | 252.922 | 115 | 10.735 | .905 | .053 | -.006 | .887 | .894 | .006 |
|  | Strict | 314.917 | 135 | 61.995 | ≥.0001 | .056 | .003 | .852 | .882 | -.035 |
